# An Experimental Evidence on Eco-Friendly Advertisement Appeals and Intention to Use Bio-Nanomaterial Plastics: Institutional Collectivism and Performance Orientation as Moderators

**DOI:** 10.3390/ijerph18020791

**Published:** 2021-01-18

**Authors:** Syed Hassan Raza, Umer Zaman, Moneeba Iftikhar, Owais Shafique

**Affiliations:** 1 Department of Communication Studies, Bahauddin Zakariya University, Multan 66000, Pakistan; hassansherazi@bzu.edu.pk; 2 Endicott College of International Studies, Woosong University, Daejeon 34606, Korea; 3 Department of Mass Communication, Lahore College for Women University, Lahore 54000, Pakistan; moneeba.iftikhar@lcwu.edu.pk; 4 Department of Islamic and Conventional Banking, Institute of Business, Management and Administrative Sciences, The Islamia University of Bahawalpur, Bahawalpur 63100, Pakistan; owais.shafique@iub.edu.pk

**Keywords:** eco-friendly advertising, bio-nanomaterial plastics, sustainable products, cultural dimensions, Global Leadership and Organizational Behavior Effectiveness (GLOBE)

## Abstract

Plastic waste management has become a serious environmental and health concern owing to large amounts of plastic deposits globally. Recently, innovative and sustainable solutions have been introduced (e.g., bio-nanomaterial plastics) to overcome the growing environmental threats. Hence, green marketers need to develop effective advertising campaigns to enhance the usage of bio-nanomaterial plastics. Past literature has suggested that cultural value-laden advertising appeals can give sustainable behavioral cues to consumers. Hence, this research unfolds the underlying cultural dimensions between the value-laden eco-friendly advertising appeals and intention to use bio-nanomaterial plastics (henceforth IBP). The present study proposes a moderating model in which two dimensions presented in the Global Leadership and Organizational Behavior Effectiveness (henceforth GLOBE) framework interact with the individuals’ perception of eco-friendly advertising appeals (henceforth IPEA) to drive bio-nanomaterial plastics usage. The model was tested by conducting an experimental study on a sample of 364 Pakistani consumers. Findings of structural equation modeling show a significant difference in the relationship between IPEA and IBP, which is moderated by the performance orientation (henceforth PO) and institutional collectivism (henceforth IC) dimensions with diverse intensity. These findings validate the effectiveness of PO and IC (as cultural dimensions) and eco-friendly advertisements that can potentially promote the consumption of bio-nanomaterials plastic.

## 1. Introduction

In the recent decade, the management of plastic-based waste has emerged as an enormous global challenge [1]. The developed countries have been managing plastic wastage by adopting smart solutions such as recycling as well as exporting the wastage to other countries having recycling facilities [2]. However, an increase in plastic wastage production compared to its recycling rate for instance, in the United States is worrying because a large number of plastic-based wastage is non-recyclable. Furthermore, China was managing the large proportion of the recycling of the global plastic waste materials, but China has barred the importation of most plastics wastage that is not equal to their fresh rigorous purity criteria. Moreover, according to the US Environmental Protection Agency, only 28–29% of the material ends up in recycling, while remaining leftover plastic wastage that cannot be exported to China is reaching South East Asia and South Asia that is ending up in landfills because the wastage contains non-recyclable plastic disposals [3]. On the other hand, a large portion of the global population is living in the developing world and lack sophisticated recycling facilities [2]. Given the increase in growing plastic waste, issues like the dumping of plastics in landfills and growing carbon footprints are emerging [2]. For instance, globally large cities, particularly in South Asia, lack recycling facilities; that leads to the dumping of waste in landfills, including plastic-wastage [3]. Owing to this problem, plastic wastage is accumulating on roadsides in some instances. The condition of a shortage of recycling facilities and underprivileged plastic waste management in developing nations such as Pakistan is becoming more threatening to environmental and human health [3]. Pakistan, being the world’s 6th most inhabited nation, is producing more plastic wastage compared to the number of amenities available to safely handle it. As a result of a lack of recycling and other viable eco-friendly waste management resources, burning plastic leftover is common practice, causing harmful pollutants to be exposed. Thereby, plastic-based wastage is affecting the environment and human health in several ways. Hence there is a dire need to promote individual-level usage of eco-friendly materials including plastics, particularly in developing nations such as Pakistan.

Recently, scientists are innovating in the development of sustainable and renewable materials to provide alternatives such as bio-nanomaterials [4]. These sustainable and renewable bio-nanomaterials can be developed through a biodegradation process involving the use of biological agents to achieve the degradation of materials [2]. Generally, microbes e.g., bacteria have been used in the biodegradation procedure to produce eco-friendly renewable raw resources [2,4]. In that way, bio-plastics have been developed with biodegraded features for substituting fossil-based plastic packing materials such as polyethylene [2]. Recent research has suggested that the integration of bio-nanomaterials with bio-plastics can further enhance the biodegradability of plastics [2]. Hence, bio-nanomaterial plastics can be utilized for environmentally friendly packaging.

Research suggests that promoting the usage of such environmentally friendly products can be attained through persuasive tools of communication such as advertising [5,6]. Hence, research on advertisement’s appeals that can persuade the consumption of products manufactured from bio-nanomaterial such as plastics can help to develop effective advertising strategies. Recent research on advertisement performance criteria has demonstrated that an adaptation strategy is more efficient for effective advertisement appeals [7,8], because consumers perform a perceptual comparison regarding the resemblance or discrepancy of reflected values in advertisement’s messages or cues (e.g., appeals) and their own set of cultural norms [8]. Greater resemblance indicates consumers observe a match among the cues (e.g., appeals) in the advertisement and their self-conceptions of acceptable cues (norm-congruent) [9]. In contrast to little resemblance, greater norm resemblance will gradient consumers to thoughtfully attend to and deliberate the advantages of the advertised product [7,8]. Hence, advertisement messages with culturally adapted value appeals are demonstrated to be more convincing and well adored by receivers than advertisement messages with lesser consistency with cultural values [7,8,9]. As a result, an understanding of culture will be viewed as increasingly important in producing effective advertisement appeals [9]. In the past decades, various cultural models have emerged in marketing and advertising. The prominent among them is Hofstede’s model which has been mostly applied in global marketing and advertising [10]. However, scholars [11] noted that the usage of Hofstede’s cultural dimensions in deciphering advertising effects on consumer attitudes and information processing is poorly understood due to the self-reported values of the cultural dimensions.

To address this issue, literature [12] recommends that there are two aspects of values conceptions that must be considered in advertisement and cultural research. The first aspect to be considered is the actual norms that reflect the advertisement that is desirable in society, while the second aspect is related to the measurement of societal-level values [11,12]. Therefore, using societal-level variables could explain an individual’s desirable behavior towards advertisement appeals [13,14,15]. Based on these recommendations [11,16], this study has applied two cultural dimensions of the GLOBE model, namely; performance orientation and institutional collectivism that is specifically relevant in the context of this study. For instance, commonly consumers do appreciate the product performance-related features. Therefore, when an individual gets exposure to the performance-oriented eco-friendly advertisement that promotes the benefits of bio-nanomaterial plastics, it would be positively evaluated by him/her based upon higher cultural orientation towards performance. The use of relevant GLOBE dimensions of PO and IC would help in providing answers to important issues like how salient cultural dimensions in Pakistan interact with eco-friendly advertising appeals to influence IBP. In sum, this experimental study examines a novel mechanism to what extent the level of advertising appeal is congruent with one’s cultural orientation that may diminish or increase the usage of sustainable products such as bio-nanomaterial plastics.

## 2. Theoretical Background and Hypothesis Development

### 2.1. Effects of Advertising Appeals on Intention to Use Bio-Nanomaterial Plastics

The Similarity Attraction Paradigm model (henceforth SAP) suggests that ad appeals embedding values robust favorable responses between individuals [17]. When individuals are exposed to communication content, SAP undertakes that they are probed for a frame of reference to form sense and interpretation. In doing so, they sense utmost reliance and temptation towards such contents which are arbitrated to be similar to their cultural values [18]. For that reason, congruity of ad appeals with the cultural background, consequently, arouses individual sensitivities of appraisal and increases the chance of favorable responses towards the given brand [19]. This suggested drawing a hypothetical model from the notions of GLOBE and SAP models that is a “match” among societal culture, the advertising appeal, and the shared individuals’ anticipations in a given culture, in predicting individual-level perceptions of advertising efficacy [14,19].

Thus, it is assumed that the IBP in response to advertising appeals relies on the individual’s cultural deciphering of appeals. However, it is expected to be stronger when the perceived level of advertising appeals depicting a particular dimension match with the culturally endorsed level of that dimension [9]. In other words, if advertising appeals do not conform to the cultural norm, advertising appeals may in turn be misinterpreted [8]. Drawing on the prior researches [9,20], different cultural values embedded in advertising appeals are said to be positively affected by the individual’s perception of the advertising appeal. Pakistani individuals shared cultural attributes of high inclination towards PO and IC [21]. Individuals normally respond to the situation/stimuli by using the norms that they uphold [22]. For example, when an individual who upholds higher PO (cultural norm) is exposed to the advertisement of a product that underlines the performance of the product, he or she is more probable to demonstrate a favorable reaction. To put it simply, the individuals develop positive perceptions towards the stimuli (advertisement) when they encounter advertisements manifested with similarities with their cultural norms [17]. Therefore, the IPEA is likely to be contingent on the extent of prominence individuals dwell on a particular dimension based on the prominence of a particular dimension in his/her society. Indeed, upheld cultural norms guide individuals to visualize their cultural preferences when they are exposed to stimuli such as advertisement [14,22]. Thereby, regardless of product benefits delineated in the advertising appeals, cultural norms can still impact advertising effectiveness. Another description of this phenomenon is also explained in literature grounded in social identity theory [23]. Thus, the individuals’ identification with a brand enables them in establishing societal locus by becoming a member of an anticipated individual in-group [17]. Recent advertising studies [11,24] have also identified that high norm congruity stimulates individuals’ identification, which intrinsically robust positive IBP. Therefore, Pakistani individuals are expected to positively respond to the eco-friendly advertising appeals wherein they found the inclusion of higher-level congruence in terms of cultural dimensions. Thus, based on the literature we hypothesize that:

**Hypothesis 1 (H1).**  *Individual perception of a higher level of PO (H1a), and IC in an Advertising Appeal (H1b) will lead to a more positive IBP.*

### 2.2. Moderation of the Performance Orientation

The performance orientation is the extent to which a human community encourages and rewards, setting challenging goals, innovation, and performance improvement [25]. PO as a cultural value is described as the need for accomplishment or as the need to constantly do better [26]. According to the literature [14], people with a higher need for accomplishment incline to acquire pleasure from constant improvement. Hence, in a high-PO society, the value is given to competitiveness and vice versa in the case of low orientation [21,25].

Past literature shows that PO has been incorporated in advertising appeals [26]. A person’s perception and appraisal of such ad appeals are highly dependent on his/her perspective. From the theoretical perspective, adaptation-level theory ALT [27] and the range frequency model (RFM) [28] assume on the earlier confronted stimulus that the individuals have a specified adaptation level, which functions as an appraisal measure for successive perceptions and appraisals of the stimulus. Regarding advertising, both theories propose that the perception of PO in ad-appeals may be reliant on the extent of an individual’s context of the PO [29]. This serves as an appraisal measure for the individual’s perception of the PO in a given advertisement [26]. This implies that in a society with an excessive appraisal measure concerning PO, eco-friendly advertisement embedding PO appeal may be regarded as a positive stimulus. Thus, the high extent of performance among Pakistanis would assist as a frame of reference. It is recommended that in a society such as Pakistan with a higher assessment standard concerning PO, advertisements designed to integrate the performance inclined appeals may be perceived positively in nature [30].

Inversely, in a cultural context that is lesser performance-oriented, where people have less reference worth concerning performance, very similar advertisements may be less regarded. Thus, the strength of the relation between the IPEA and IBP would be dependent on the culturally learned context of PO. In a nutshell, this is consistent with the adaptation-level theory and GLOBE model [25] that an individual’s judgment of a stimulus would rely on his/her prior learned experiences such as cultural orientations. Thus, the Pakistani context of high PO would moderate this link that a higher degree of PO perceived in an eco-friendly ad appeal leads towards a favorable IBP. Thus, it is hypothesized that:

**Hypothesis 2 (H2).**  *The effect of PO will be stronger among Pakistanis as individuals place greater importance on PO and thus positively moderate the relationship between IPEA and IBP.*

### 2.3. Moderation of the Institutional Collectivism

In the past, there has been a wide debate on the construct of collectivism in anthropology’s value theory [31], psychology [32,33], and sociology [34]. Across all of these areas, the landscape of the association among the individual and the society remains a central concern, which is largely stated as individualism/collectivism. This notion has also been mentioned to be self-emphasis and collectivism. Despite the elusive abstract discrepancies of these terminologies used by several scholars [31,33], the central idea was to define the level to which individuals are self-directed or their actions are entrenched with societal goals. The GLOBE project greatly elaborated (IC) as “the degree to which organizational and societal institutional practices encourage and reward collective distribution of resources and collective action” [25], (p. 23). Institutional collectivism is a sense of collective efforts and actions to achieve collective future goals [15]. Individuals of the culture high on IC believe in cooperation and integration into the group.

From the persuasion theory standpoint, the similarity is the influential principle of persuasion because the similarity (i.e., resonant cultural cues in an ad) surges likeability [18]. Scholars [35] noted that such likeability can be triggered through a scant perception of resemblance in given stimuli (e.g., eco-friendly ads). Thereby in literature, similarity has been regarded as an imperative source and principle of persuasion [17]. This similarity attraction paradigm (SAP) phenomenon can be explained as individuals are more fascinated by stimuli (whether source or content) that are similar to their perspective or experience [36]. The SAP has been supported in the Asian context, for instance, Chinese customers favor ad incorporating culture and such a cultural converging advertising strategy is found to be effective [37]. Similarly, Asians are found to be inclined towards the sources on similar cultural values [38,39]. These results demonstrate that when people from collectivistic cultures such as Pakistan observe advertisements incorporating appeals similar to their cultural orientation, they have a positive response.

Asian countries are ranked highly as collectivistic societies; thus, advertisers use collectivistic appeals in Asian countries [40]. Past studies have only used Hofstede’s dimension in studies such as green ad appeals with collective orientation [41], global brands [40], and corporate social responsibility [42]. Past studies have revealed that people are favorable towards collectivistic appeals highlighting national interests such as environmental concerns [43,44].

Thus, collectivistic culture like Pakistan has an expressive common emphasis on collectivism and people are more anxious about group desires; hence, they prefer collective goals. Collectivistic cultures such as Pakistan have shared principles and what matters is the collective goal of their group rather than individual gain [21]. The alarming situation relating to landfill shortage and plastic wastage demerits for a common person’s health is a collective concern [2]. In this regard, eco-friendly advertising revealing the usage of bio-nanomaterial plastics as a collective gain could be appealing for Pakistanis based on their underlying cultural value. Therefore, building on the SAP model [17], Pakistanis evaluate the embedded appeal of IC by using their cultural orientation of IC in determining their responses towards bio-nanomaterial plastics usage. In this way, the cultural orientation of IC serves as the moderating factor, and individuals exposed to eco-friendly ads embedding IC appeal/cues (that is, use of eco-friendly plastics are a collective gain as it saves the environment) matching with their cultural prefaces of IC leads towards positive IBP in response. Thus, the strength of ad appeals and IBP relation depends on the level of congruence with the cultural orientation of the IC and it is hypothesized that:

**Hypothesis 3 (H3).**  *The effect of IC will be stronger among Pakistanis as individuals place greater importance on IC and thus positively moderate the relationship between IPEA and IBP.*

**Figure 1:**
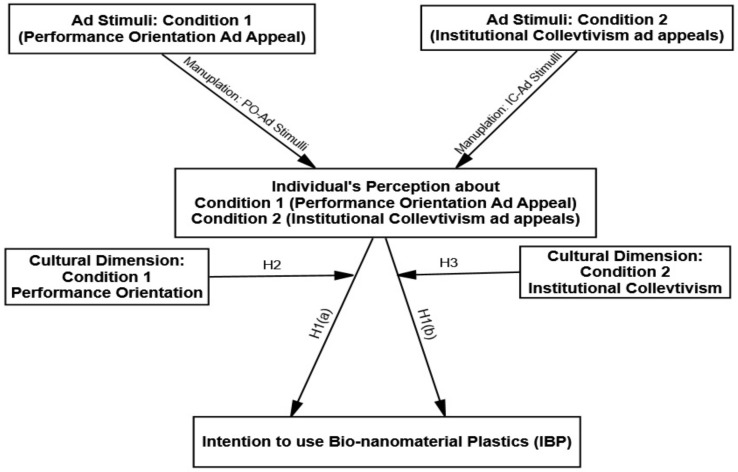
Proposed Conceptual Model.

Based on the review of mainstream literature, the hypothesized relationship in a conceptualized model of intentions to use bio-nanomaterial plastics is presented in Figure 1.

## 3. Materials and Methods

### 3.1. Participants and Procedures

The present study proposes to underpin the moderating effect of cultural values upheld by individuals in predicting the relationship between eco-friendly advertising appeal embedded cultural dimensions on the IBP. The fundamental idea behind the study is that a given eco-friendly advertising appeal is expected to be perceived and assessed in cultural context; the appeal relies on the degree of importance of individuals’ dwelling on a particular dimension (e.g., individual-level IC) along with the level of a particular dimension in the society in which the individual belongs (e.g., societal-level IC). Henceforth, the study clearly distinguishes among cultural dimensions on an individual level vs. cultural dimensions on a societal-level by using two-facet constructs of GLOBE as well as eco-friendly ad-stimuli reflecting/embedding the cultural dimension.

To validate this in line with past researches [16,45], an experimental design is employed so that participants can be exposed to the eco-friendly advertising appeals embedded with the given advertising appeal. After viewing the eco-friendly advertisement, participants’ responses would be based upon their culturally learned level of importance that the individuals place on a particular dimension. This study used a sample of 364 Pakistani nationals. For the recruitment of the participants, an invitation was sent to the participants by using several means like emails, announcements on social media, and with the assistance of the volunteers.

The participants were requested to gather at the on-campus facility. The participants were informed about the purpose of the study and asked to sign a consent to participate in the experiment. The 364 participants considered for the study were randomly assigned to the two experimental conditions (n = 182). Therefore, participants were exposed to the two different eco-friendly ad-stimulus (ad-stimuli for one dimension) and experimented. After the exposure of the relevant eco-friendly ad-appeal participants were asked to fill the questionnaire to tap IBP as a result of the exposed eco-friendly ad-appeal (PO ad-appeal etc.). The sample size in this study is common for experimental design and in advertising research underpinning cultural values and ad-appeals [8,17].

### 3.2. Instrumentation

#### 3.2.1. Ad-Stimuli

Two advertisements were used as the stimuli; these eco-friendly ads represented a high level of cultural orientation (that is, PO and IC). To ensure relevant selection, the eco-friendly ad-stimuli and other instruments were subsequently pretested in two phases of the pilot studies. In the first phase, a pilot study was carried out with a group of 30 students at a major university in Pakistan. After reading the advertisements, the participants were asked to fill a questionnaire that involved queries on how much they perceived the advertisements as congruent with the dimensions (i.e., performance-oriented). Changes were incorporated to improve the stimuli to achieve the desired level. In the second phase, comments from 5 advertising professors and 5 experts from advertising agencies were also solicited. Minor amendments were made to improve the clarity and relevance of the questionnaire. These pre-tests ensured that the experimental procedures and ad-stimuli were well understood.

#### 3.2.2. Individual’s Perception of Eco-Friendly Advertising Appeals (IPEA)

This study adopted and modified the 3-items scale of measuring individual perception of PO ad appeals from the literature [30] the seven-point Likert scale: “1 = strongly disagree 7 = strongly agree”. Participants were asked to evaluate the product used in the eco-friendly ad. The items read: “The bio-nanomaterial plastic product in the advertisement seems to me to be …” (1) “performance-oriented”, (2) “better than existing plastic products”, and (3) “beneficial”. In order to measure the individual perception of IC ad appeals, the 3-items scale was used as derived from the GLOBE’s definition of the IC dimension: “The advertisement of bio-nanomaterial plastic seems to me representing…” (1) “Collective achievement”, (2) “concerns about environmental issues of my nation”, and (3) “group loyalty”.

#### 3.2.3. Cultural Dimensions

This study adopted the items of measuring two cultural dimensions based on the conceptualization of the GLOBE [25,30], 4 items for PO were read as: “In Pakistan people generally (1) “… strive to continually improve their performance”, (2) “… reward those who strive for performance and innovation”, (3) “… set challenging goals for themselves”, and (4) “… base rewards on effective performance”. Likewise, 4 items for IC from the literature [25] their statements were: “In Pakistan people generally (1) “… encourage the group loyalty even if individual goals suffer”, (2) “… encourage group loyalty”, (3) “… maximize individual interest than the collective interest (reversed score)”, (4) “… collective interest than the individual interest”, and a seven-point Likert scale was used: 1 = strongly disagree to 7 = strongly agree.

#### 3.2.4. Intention to Use Bio-Nanomaterial Plastics (IBP)

This study used a total 5-items scale of measuring IBP from the literature [46,47] on the seven-point Likert scale: “1 = strongly disagree 7 = strongly agree”. The scale is used to tap the social influences alongside the purchasing aspect which are two important domains in the context of this study. The same scale was used to measure the IBP in responses about the appropriate ad appeal (i.e., PO and IC) with bit language alterations. The items used to measure IBP: (1) “When buying plastic product next time, I predict that I will choose a product using bio-nanomaterials”, (2) “I am willing to use bio-nanomaterial plastic products”, (3) “Given the information in the advertisement, I am willing to buy bio-nanomaterials plastic products”, and (4) “I predict if I buy bio-nanomaterials plastics it will beneficial for collective goals of my nation’s environmental protection (for IC), but (for PO) modified and words “for collective goals of my nation” was deleted, and (5) “I always buy the product [plastic] after thinking much about its “benefits for my society” (for IC) and “performance” (for PO).

#### 3.2.5. Control Variables

This study measured the socio-demographic characteristics namely; (1) sex, (2) education, (3) income, and (4) age as the control variables (see Table 1).

## 4. Results

### 4.1. Preliminary Analysis

The descriptive analysis to screen the normality of the data was performed. The data revealed the normality based on the cut-off values of the recommended normality test and visual inspections of all constructs after deleting the outliers. The main analysis comprises n = 356 and n = 178 for each group after the deletion of the cases. Before proceeding, bivariate analysis was conducted separately for two assumed models containing the relevant ad-appeal (for example, PO and IC) construct, cultural dimension, and IBP (see Table 2).

Additionally, an assessment of multicollinearity between the predictor variables was carried out by using the variance inflation factor (VIF) as well as the tolerance index (TI). Both VIF and TI were found to be within the acceptable threshold for collinearity (VIF is > 10 and TI > 0.84).

The confirmatory factor analysis (CFA) was conducted using AMOS 23.0 to analyze the uniqueness of all constructs including cultural dimensions IPEA, IBP, PO, and IC.

In total, four models based on all the possible factors (structure) were examined concerning the constructs of IPEA, IBP, PO and IC that include two separate models for each dimension: (a) One single-factor model, one for each dimension’s data by loading items of IPEA, IBP and the dimension in question on a single-factor to check CMV; and (b) one model of the three-factor models, by loading items of the IPEA, IBP, and the dimension in question on their parent factor. The results of the CFAs in Table 3 clarified that when all items of IPEA, IBP, PO and IC are loaded on their parent factors, use the best fit among all other hypothesized models. All items loadings (see Table 4) on their parent factors were significant; the results showed consistent factor structure and model fitness.

Information related to the composite reliability, Cronbach’s alpha and average variance extracted for each variable is available in Table 5. Furthermore, the discriminant validity of the constructs is within the acceptable ranges from the results of each dimension (see Table 5 for details) and suggests advancing with hypotheses testing.

### 4.2. Hypothesis Testing

For testing the moderation of cultural norms, in total, four models were examined (two for each dimension). To do so, Model 1 examined the main effect of an individual’s perception of eco-friendly advertising appeals and cultural dimensions (PO and IC). To evaluate the possible influence of the control variable, a stepwise analysis approach was adopted. In this recommended approach all control variables of this study, namely, sex, age, income, and education were added in Model 1. However, based on the findings the null hypothesis of the control variables impact was rejected.

Model 2 was used to examine the moderation effect of the PO and IC. Next, the slope was examined for the main effects of IPEA and PO as random effects. For IBP, in the case of the PO data set, the main effect of IPEA is (*β* = 0.32, *p* < 0.001) and PO is also significant (*β* = 0.44, *p* < 0.001) and 52% of the variance was recorded for IBP. Therefore, H1 (a) is supported, as was expected that IPEA would be more positively related to IBP among Pakistanis (see Table 6).

The moderation of PO Model 2 among sample also revealed the interaction of IPEA and PO and described 67% variance for IBP with an adequate model fitness: χ2 = 837.14, df = 97, χ2/df = 2.82; CFI = 0.94; IFI = 0.96; TLI = 0.93; SRMSR = 0.063; and RMSEA = 0.03. The model showed consistency with model fitness cut-offs [48,49]. The change in the R^2^ generated by the interaction term of PO and IPEA (see Table 5) was within the suggested range for moderating effects, which suggested that the impact of IPEA on IBP varied as a function of the PO. The interaction effect between IPEA and PO was also significant (*β* = 0.14, *p* < 0.001).

**Figure 2:**
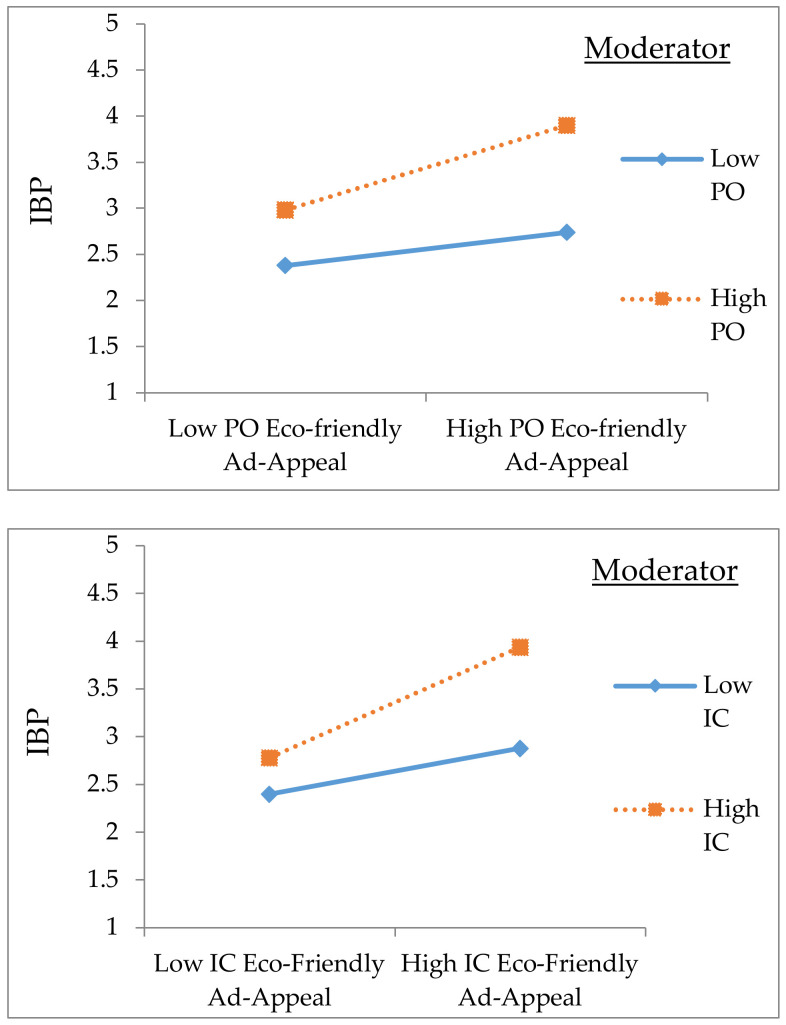
Moderation of the PO and IC dimensions.

Furthermore, the simple slopes were examined for analyzing the nature of the IPEA × PO) interaction [50,51]. The simple slopes were examined with the high PO (1 SD higher) and low PO (1 SD higher; see Figure 2). Our results support H2 as there is a positive relationship between IPEA and IBP was strong in the presence of PO (*β* = 0.14, *p* < 0.001, R2 change = 0.15). It can then be concluded that, in the case of eco-friendlily advertising appeals and PO cultural value congruence, Pakistanis would have more IBP. Equally, the same approach was implemented for testing the moderation of the IC dimension. The main effect of IPEA on IBP was (*β* = 0.41, *p* < 0.001), and IC was (*β* = 0.36, *p* < 0.001) and H1(b) is supported. The interaction effect between IPEA and IC was (*β* = 0.17, *p* < 0.03) with R^2^ = 0.61. This supports H3; therefore, it is concluded that in the case of matched perceived level of IC eco-friendly advertising appeal with the individuals’ level of IC value, Pakistanis would have a more positive relationship between IPEA and IBP.

## 5. Discussion

This experimental study explains the effect of PO and IC manifested eco-friendly advertising appeals to understand the consumer decision on bio-nanomaterial plastic products. The current study addresses the novel question how similarity with consumers uphold cultural values PO and IC with the stimuli in question could influence consumers’ decisions on bio-nanomaterial plastic products. To do so, two separate experimental groups were selected and were exposed to two different ad stimuli containing PO and IC appeals. The empirical finding of CVB–SEM indicates that advertisements manifesting PO and IC, EC has a robust effect on IBP in Pakistan, and supported H1(a) (*β* = 0.32, *p* < 0.001) and H1(b) (*β* = 0.41, *p* < 0.001). These findings validate the past research that relates advertisement effectiveness with the cultural deciphering [18,21,52,53,54]. However, the intensity of these effects suggests an interesting pattern among Pakistani prospective consumers of bio-nanomaterial plastics. Instead of an apparent rational appeal of PO, Pakistanis were found more persuaded by the affective appeals IC. For that reason, it supports past green marketing studies conducted in the Asian context that suggested the purchasing decisions of Asian consumers can be influenced by emotional motivational factors such as the “welfare of the nation” [6,55].

Outcomes from past studies [17,18,19,20,21], confirmed the involvement of cultural factors in predicting the effectiveness of advertising in diverse areas. The past researches remained inconclusive in many instances, while integrated the environmental awareness as consumers. For example, when scholars applied environmental knowledge and concerns found no interaction and on some occasions the insignificant relationship was found in a different culture. Therefore, advertising scholars suggested [56,57] to systematically test the advertisement effectiveness on behavioral outcomes in the cultural framework of the GLOBE. In this regard, this paper addresses how cultural dimensions (defined as in the GLOBE framework) moderate the effectiveness of eco-friendly advertising appeals by embedding values of PO and IC that drives IBP. Thus, two hypotheses H2 and H3 to explain whether IBP and IPEA were posed to find out the moderating effect of cultural dimensions PO and IC regarding a sustainable product. Moderation analysis using SEM revealed that cultural norms congruence among the Pakistanis moderated the association of IPEA and IBP. Further slope analysis [50] also confirmed that Pakistanis have a more positive IBP when advertising appeal is culturally congruent. In general, these findings validate that consumers’ decisions are not straightforward and involve a complex mechanism by which they are culturally deciphered. In this regard, the message familiarity with their cultural orientations can activate positive evaluations of the sustainable products in question. These results support the notion that cultural norms have an imperative role in the formulation of consumer decisions related to eco-friendly or sustainable products.

### 5.1. Theoretical and Managerial Implications

In the 21st century with increased transnational information flow, predicting, and clarifying differences in consumer behavior across cultures has become indispensable [11]. Practicing advertising to expand the business or promoting the social cause to nations with different cultures and societal norms without tailoring advertisements to their cultural norms can prime the result to serious losses [58]. Gradually, albeit, researchers realize that there are several consumption differences across cultures because individuals are often not rational and do not make buying decisions that maximize utility [12]. Scholars [22,56] argued that consumer practices may be found in the global marketplace, but underlying norms that explain intentions for purchasing these are not global. Thus, the notion of rationality is gradually observed as impractical and seats individuals outside of a cultural context [59]. Overlooking culture’s impact has led several corporations to integrate marketing, which instead of growing effectiveness caused lessening success [60]. Numerous large advertising campaigns resulted in a decline in profits because such integrated marketing operations lack local cultural sensitivity [24]. For instance, diminishing profitability led Coca-Cola to manifest local cultural values in their advertising campaigns. Empirical studies using national cultural dimensions are also made it evident that instead of using standardized practices, to understand the local culture sensitivity had become undeniably vital to success [59]. This entails that effective advertising needs to understand the distinctiveness of consumers’ cultures.

Research has provided ample evidence that the success of advertising or promotional campaigns can be forecasted by considering cultural context in advertising campaigns [11,38,56]. For instance, several cultural attributes, future orientation [61], performance orientation [30], and gender egalitarianism [13] have been identified in predicting advertising effectiveness. These cultural attributes defined in the cultural dimensions such as GLOBE were substantiated to be theoretically valuable in quantifying advertising effectiveness. This is consistent with the view of scholars [24,30,59] that an individual of any culture perceives advertising by using their cultural attributes. In this standard, advertisers must know that their product does not automatically show the same effects in culturally dissimilar nations as the cultural effects have more implications [58,62]. In this regard, a stream of literature has called for more researches assessing nations’ cultural norms influence to delineate the advertising effectiveness. For advertising, it is desired to understand the features of the cultures because they influence the behavior of individuals [13,61].

This paper contributes to the on-going discussion about the influence of cultural norms in advertising literature by using the two dimensions of the GLOBE framework to operationalize certain cultural norms [7,13,39,52]. A careful examination of the recent literature raises some inspiring new queries and issues in measuring cultural influences [13,39,41,43,61]. First, investigations supporting such cultural influences most often have adopted a conventional ecological approach by only measuring the individual’s preferences and therefore was not strong enough in generalizing for other factors [14,52]. This study uses the culture level analysis instead of a value and practice-based onion approach. In taking an onion assumption approach, the cultural values are expected to drive cultural practices [8,29]. The current study addresses this question and sheds innovative light on how cultural dimensions influence the consumers’ decisions related to bio-nonmaterial plastic usage. Lastly, this study is undertaken in adherence to the call for research tapping into concepts that can connect individual-level behavioral outcomes with their societal-level causes such as environmental issues [41,63]. It explains many other diverse cultural influences such as PO which may drive eco-friendly behavioral patterns of the individual. This approach helps theoretical testing of the influence of culture in explaining what is significant or desirable for individuals in Pakistan.

The evidence from this study will help ad practitioners to determine whether the attribution of appeals in advertisements from GLOBE dimensions is effective in developing promotion strategies related to environmental issues. Advertisers promoting sustainable products may consider PO and IC appeals in eco-friendly advertisements. But results found that emotional and collective benefits have more stringent effect than the apparent rationale ad message delineating the bio-nanomaterial product’s self-benefit. Ergo, managers in Asian countries may devise future eco-friendly advertisements and eco-labels delineating their usage benefit for the society. In the context of bio-nanomaterials, it would be useful to inform the public how it is helpful for their nation’s welfare. For example, in Pakistan, the plastic waste management situation is alarming, posing collective health and environmental risks [3]. In this scenario, managers must focus on the collective benefits of the bio-nanomaterial along with the self-benefit of its usage for the individuals. Hence it can be concluded that the study validates the similarity paradigm theories [17,23,27,34,35] that convergence is an inevitable idea in practicing advertising effectiveness. In other words, regardless of product benefits delineated in the advertising appeals, cultural orientations incorporated in eco-friendly ad appeals can still impact advertising effectiveness. In this standard, the usage of cultural dimensions (e.g., PO and IC) can benefit advertisers to modify the consumers’ intentions towards buying sustainable products.

### 5.2. Limitations and Future Directions

This experimental study is conducted to fully understand the intervention of advertising appeal on intention in the context of bio-nanomaterial plastics usage in an Asian nation. Still, there are some restrictions based on the nature of the sample. Therefore, future research may consider other ethnic or gender diversity within samples to replicate the proposed model. Future studies may consider the cross-national comparison to further validate the effectiveness of the cultural dimensions’ usage in eco-friendly advertisements. Another limitation of the study is related to the selection of constructs; future studies may include some other important constructs such as cost, perceived threats, etc. This would give insight that whether cultural dimensions drive the environmental concerns which can lead towards more positive intention to use sustainable products. Similarly, the influences of other forms of communication contents (e.g., eco-labels) can be examined. This would give helpful directions to managers interested to promote sustainable products.

## Figures and Tables

**Table 1:** Demographic Characteristics.

Demographic	Frequency	Percentage
**Sex**		
Male	216	59.3
Female	148	40.7
Education		
Primary School	28	7.7
High School (Matriculation)	95	26.1
Undergraduate	162	44.5
Master and Above	79	21.7
Income (Pakistan Rupees)		
Low Income (10,000–30,000)	119	32.7
Middle Income (31,000–75,000)	162	44.5
High Income (76,000–above)	83	22.8
Age Group		
18–30	187	51.4
31–44	109	29.9
45–above	68	18.7

**Table 2:** Mean, Standard Deviation, and Correlation.

**PO –Ad Appeal**	**Mean**	**IPEA**	**IBP**	**PO**
IPEA	4.26	1		
IBP	4.39	0.49 *	1	
PO	4.81	0.41 *	0.54 *	1
**IC –Ad Appeal**	**Mean**	**IPEA**	**IBP**	**IC**
IPEA	4.32	1		
IBP	4.40	0.32 *	1	
IC	4.34	0.35 *	0.46 *	1

* *p* < 0.01. IPEA = individuals’ perception of eco-friendly advertising appeals, PO = performance orientation, IC = institutional collectivism, and IBP = intention to use bio-nanomaterial plastics.

**Table 3:** Confirmatory factor analysis.

Models	χ2	df	χ2/df	GFI	TLI	IFI	CFI	RMSEA
PO –Ad Appeal	589.11	412	1.43	0.93	0.95	0.96	0.96	0.032
IC –Ad Appeal	567.38	341	1.66	0.94	0.96	0.97	0.97	0.030

PO = performance orientation, IC = institutional collectivism, χ2 = chi-squared, df = degree of freedom.

**Table 4:** Standardized loadings.

	Indicators	PO (Model)	IC (Model)
Variables		Loadings	Loadings
Individuals’ perception Eco-friendly Ads	IPEA1 (PO)	0.80 *	
	IPEA2 (PO)	0.87 *	
	IPEA3 (PO)	0.92 *	
	IPEA1 (IC)		0.86 *
	IPEA2 (IC)		0.78 *
	IPEA3 (IC)		0.75 *
	IBP1 (PO)	0.83 *	
	IBP2 (PO)	0.63 *	
	IBP3 (PO)	0.75 *	
	IBP4 (PO)	0.78 *	
	IBP5 (PO)	0.67 *	
	IBP1 (IC)		0.69 *
	IBP2 (IC)		0.73 *
	IBP3 (IC)		0.68 *
	IBP4 (IC)		0.76 *
	IBP5 (IC)		0.83 *
*Cultural Dimension: PO*	PO1	0.69 *	
	PO2	0.83 *	
	PO3	0.71 *	
	PO4	0.89 *	
*Cultural Dimension: IC*	IC1	0.66 *	
	IC2	0.72 *	
	IC3	0.65 *	
	IC4	0.70 *	

IPEA = Individuals’ perception of eco-friendly advertising appeals, PO = performance orientation, IC = institutional collectivism, IBP = intention to use bio-nanomaterial plastics, * = significant loadings.

**Table 5:** Discriminant and Convergent Validity.

**PO –Ad Appeal**	**α**	**CR**	**AVE**	**IPEA**	**IBP**	**PO**
IPEA	0.83	0.91	0.75	(0.86)		
IBP	0.72	0.85	0.54	0.21 *	(0.73)	
PO	0.77	0.88	0.62	0.27 *	0.25 *	(0.78)
**IC –Ad Appeal**	**α**	**CR**	**AVE**	**IA**	**IG**	**IC**
IPEA	0.89	0.82	0.63	(0.79)		
IBP	0.82	0.86	0.55	0.31 *	(0.74)	
IC	0.76	0.83	0.54	0.16 *	0.29 *	(0.73)

* *p* < 0.05, CR = composite reliability, AVE = average variance extracted.

**Table 6:** Moderation of cultural dimensions results.

Stepwise Moderation	Model 1	Model 2
Dependent Variables: IBP		
Step 1: Independent Variables: IPEA and Control Variables	0.32 * (2.63)	0.32 * (2.63)
PO	0.44 * (5.12)	0.44 * (5.12)
R^2^ Step 2: Moderator IPEA × PO		0.52
		0.14 * (2.32)
R^2^		0.67
ΔR2		0.15
Dependent Variables: IBP		
Step 1: Independent Variables: IPEA and Control Variables	0.41 * (3.12)	0.41 * (3.12)
IC	0.36 * (4.23)	0.36 * (4.23)
R^2^ Step 2: Moderator IPEA × IC		0.52
		0.17 * (2.49)
R^2^		0.61
ΔR2		0.09

Note: The values in parentheses represent t statistics. Entries are random effects with a robust standard error. R^2^ = proportion of variance explained by antecedent in both model 1 and 2, * = *p* is significant.

## Data Availability

The data that support the findings of this study are available from the corresponding author upon reasonable request due to ethical and privacy restrictions.
